# Impaired glucose utilization in the brain of patients with delirium following hip fracture

**DOI:** 10.1093/brain/awad296

**Published:** 2023-09-02

**Authors:** Irit Titlestad, Leiv Otto Watne, Gideon A Caplan, Adrian McCann, Per Magne Ueland, Bjørn Erik Neerland, Marius Myrstad, Nathalie Bodd Halaas, Christian Thomas Pollmann, Kristi Henjum, Anette Hylen Ranhoff, Lene B Solberg, Wender Figved, Colm Cunningham, Lasse M Giil

**Affiliations:** Department of Clinical Medicine, University of Bergen, 5020 Bergen, Norway; Neuro-SysMed, Department of Internal Medicine, Haraldsplass Deaconess Hospital, 5009 Bergen, Norway; Oslo Delirium Research Group, Department of Geriatric Medicine, Oslo University Hospital, 0424 Oslo, Norway; Oslo Delirium Research Group, Department of Geriatric Medicine, Oslo University Hospital, 0424 Oslo, Norway; Institute of Clinical Medicine, University of Oslo, 0318 Oslo, Norway; Department of Geriatric Medicine, Akershus University Hospital, 1478 Lørenskog, Norway; Department of Geriatric Medicine, Prince of Wales Hospital, 2031 Sydney, Australia; Prince of Wales Clinical School, University of New South Wales, 2031 Sydney, Australia; Bevital AS, 5021 Bergen, Norway; Bevital AS, 5021 Bergen, Norway; Oslo Delirium Research Group, Department of Geriatric Medicine, Oslo University Hospital, 0424 Oslo, Norway; Department of Internal Medicine, Bærum Hospital Vestre Viken Hospital Trust, 1346 Gjettum, Norway; Oslo Delirium Research Group, Department of Geriatric Medicine, Oslo University Hospital, 0424 Oslo, Norway; Institute of Clinical Medicine, University of Oslo, 0318 Oslo, Norway; Department of Orthopedic Surgery, Akershus University Hospital, 1478 Lørenskog, Norway; Oslo Delirium Research Group, Department of Geriatric Medicine, Oslo University Hospital, 0424 Oslo, Norway; Institute of Clinical Medicine, University of Oslo, 0318 Oslo, Norway; Department of Clinical Science, University of Bergen, 5020 Bergen, Norway; Geriatric Unit, Clinic of Medicine, Diakonhjemmet Hospital, 0319 Oslo, Norway; Division of Orthopaedic Surgery, Oslo University Hospital, 0424 Oslo, Norway; Institute of Clinical Medicine, University of Oslo, 0318 Oslo, Norway; Orthopaedic Department, Bærum Hospital, Vestre Viken Hospital Trust, 1349 Gjettum, Norway; School of Biochemistry & Immunology, Trinity Biomedical Sciences Institute and Trinity College Institute of Neuroscience, Trinity College Dublin, D02 R590 Dublin, Ireland; Neuro-SysMed, Department of Internal Medicine, Haraldsplass Deaconess Hospital, 5009 Bergen, Norway; Department of Clinical Science, University of Bergen, 5020 Bergen, Norway

**Keywords:** acetoacetate, β-hydroxybutyrate, lactate, branched-chain amino acids, 3-hydroxyisobutyrate

## Abstract

Alterations in brain energy metabolism have long been proposed as one of several neurobiological processes contributing to delirium. This is supported by previous findings of altered CSF lactate and neuron-specific enolase concentrations and decreased glucose uptake on brain-PET in patients with delirium. Despite this, there are limited data on metabolic alterations found in CSF samples, and targeted metabolic profiling of CSF metabolites involved in energy metabolism has not been performed.

The aim of the study was to investigate whether metabolites related to energy metabolism in the serum and CSF of patients with hip fracture are associated with delirium.

The study cohort included 406 patients with a mean age of 81 years (standard deviation 10 years), acutely admitted to hospital for surgical repair of a hip fracture. Delirium was assessed daily until the fifth postoperative day. CSF was collected from all 406 participants at the onset of spinal anaesthesia, and serum samples were drawn concurrently from 213 participants. Glucose and lactate in CSF were measured using amperometry, whereas plasma glucose was measured in the clinical laboratory using enzymatic photometry. Serum and CSF concentrations of the branched-chain amino acids, 3-hydroxyisobutyric acid, acetoacetate and β-hydroxybutyrate were measured using gas chromatography-tandem mass spectrometry (GC-MS/MS).

In total, 224 (55%) patients developed delirium pre- or postoperatively. Ketone body concentrations (acetoacetate, β-hydroxybutyrate) and branched-chain amino acids were significantly elevated in the CSF but not in serum among patients with delirium, despite no group differences in glucose concentrations. The level of 3-hydroxyisobutyric acid was significantly elevated in both CSF and serum. An elevation of CSF lactate during delirium was explained by age and comorbidity.

Our data suggest that altered glucose utilization and a shift to ketone body metabolism occurs in the brain during delirium.

## Teaser

Altered brain energy metabolism has long been suspected in delirium, but CSF analyses have been lacking. Titlestad *et al.* now show that delirium is indeed associated with changes in energy metabolism in the brain but not systemically. In the brain, altered glucose utilization is accompanied by a shift to ketone body metabolism.

## Introduction

Delirium is an acute neuropsychiatric disorder of attention and cognition, frequently precipitated by acute illness, trauma and/or surgery.^1–4^ Studies conducted more than 60 years ago showed that experimental hypoxia and hypoglycaemia lead to delirium-like clinical and EEG findings.^5^ The findings of elevated concentrations of CSF lactate and decreased neuron-specific enolase during delirium^6^ have suggested that cerebral metabolic insufficiency may potentially underlie delirium. This was further supported by findings of glucose hypometabolism in the brain using ^18^F-fluorodeoxyglucose (FDG)-PET in humans with delirium.^7–9^ Experimentally, hypoglycaemia was sufficient to trigger acute working memory disruption in a mouse model of inflammation-induced delirium, and glucose supplementation mitigated these delirium-like changes.^10^

Diabetes mellitus and elevated glycated haemoglobin (HbA1c) are independent risk factors for postoperative delirium.^11–13^ However, hyperglycaemia unrelated to diabetes mellitus sometimes occurs during stress and critical illness^14^ as part of an adaptive response to provide fuel to the immune system in stressful situations.^15,16^ Some studies report elevated blood glucose in delirium,^14,17^ although differences in CSF glucose concentrations are yet to be detected.^6,10^

Despite a long-standing belief that brain energy deficiency may occur in delirium, more detailed metabolic profiling of relevant CSF metabolites has not been performed. Brain insulin resistance, a condition wherein insulin fails to regulate brain metabolism,^18^ has been linked to cognitive dysfunction in several animal models, including models of Alzheimer’s disease.^19,20^ Branched-chain amino acids (BCAAs), leucine, isoleucine, valine and the valine-degradation product 3-hydroxyisobutyric acid (3-HIB) are linked to insulin resistance.^21,22^ Furthermore, if cells cannot access or use glucose, they may increase their reliance on ketone bodies such as acetoacetate (AcAc) and β-hydroxybutyrate (β-HB), derived from the β-oxidation of fatty acids.^23^ These are typically produced and released by the liver and taken up by the brain but may, under certain circumstances, also be made in the brain.^24,25^ Significantly, these metabolites can be measured in both the blood and CSF.

The aim of this study was to investigate the concentrations of metabolites related to insulin resistance and energy metabolism in the serum and CSF of patients with hip fracture, and to assess whether they were associated with delirium.

## Materials and methods

### Study participants

The study comprised two prospective Norwegian cohorts of hip fracture patients with CSF samples, representing a total of 450 patients. Owing to logistical challenges in collecting serum samples at the same time as CSF (i.e. due to limited available health personnel that could obtain the blood samples within a limited time frame), only 238 of the patients had concomitant serum samples. Twenty-five of these patients had subsyndromal delirium and therefore were excluded from the main analysis (see next paragraph). Hence, 213 serum samples were available for this study (six samples were obtained from the first cohort and 207 from the second cohort). The first cohort included 104 patients recruited from the Oslo University Hospital between 2009 and 2012. The second cohort was a multicentre study that recruited 346 patients between 2016 and 2019 from Oslo University Hospital, Diakonhjemmet Hospital, Akershus University Hospital and Baerum Hospital. All patients acutely admitted to the hospital for surgical repair of a hip fracture were eligible to participate in the study. For the first cohort, patients were excluded if the hip fracture was caused by a high-energy trauma (defined as a fall from higher than 1 m) or if they were moribund (defined as a condition where the patients were dying or considered to be too frail to survive hip fracture surgery) on admission.^26^ In both cohorts, only patients undergoing surgery in spinal anaesthesia were eligible for inclusion as the CSF was collected at the onset of spinal anaesthesia.

Participants with subsyndromal delirium can be classified as either cases or controls in biomarker studies.^27^ Therefore, 44 participants with subsyndromal delirium were excluded from the primary analyses but assessed in *post hoc* analyses (Supplementary material). Subsyndromal delirium was defined as patients with several symptoms of delirium who did not fulfill diagnostic criteria, and the procedure for diagnosing subsyndromal delirium is described in the Supplementary material. Thus, 406 participants were included in the main analyses.

### Ethics

Informed consent was obtained from all patients participating in the studies or, in the case of cognitive impairment, from a proxy. The study was approved by the Norwegian Regional committee for medical and health research ethics (REK 2016/1368 and REK 2009/450).

### Data collection

Data regarding pre-fracture dementia, diabetes and clinical status were collected during hospitalization, while data regarding delirium status were collected prospectively. Furthermore, in both cohorts, delirium was assessed daily during hospitalization (preoperatively and until the fifth postoperative day). For the first cohort, delirium was evaluated by trained nurses or physicians using the Confusion Assessment Method (CAM).^28^ The CAM was scored based on an interview with the participants, supplemented by information from relatives and nurses, as described previously.^26^ Additionally, two experienced delirium researchers (L.O.W. and B.E.N.) independently examined hospital records to reveal potential episodes of delirium. In the second cohort, delirium was evaluated using the Diagnostic and Statistical Manual of Mental Disorders 5 (DSM-5) criteria,^2^ based on a standardized procedure described previously.^29^ The same researchers as for the first cohort (L.O.W. and B.E.N.) independently assessed all available information for each patient to decide whether or not the DSM-5 criteria for delirium were fulfilled. The interrater agreement upon delirium diagnosis was excellent (kappa 0.97), with disagreements resolved through discussion. The first and second cohorts used two different assessment methods for delirium (CAM and DSM-5), as during the inclusion period of the first cohort, the CAM was the most used instrument for assessing delirium and DSM-5 criteria had not yet been published.

Dementia as defined for the purposes of this study (not to clinically diagnose dementia) was assessed using the Informant Questionnaire on Cognitive Decline in the Elderly (IQCODE), with a cut-off at ≥3.44 indicating chronic cognitive impairment.^30^ Hospital records were used for evaluating pre-fracture dementia in cases (*n* = 30) of missing IQCODE data (e.g. previous diagnosis, information from previous hospital admissions or assessments at outpatient clinics or reports from nursing homes and/or home nurses). Diabetes or the use of anti-diabetic medication was registered from electronic medical records by reviewing admission notes and discharge summaries. Furthermore, we used the preoperative American Society of Anesthesiologists (ASA) physical status classification^31^ as a proxy measure of comorbidity.

### CSF and blood sampling and biochemical analyses

Serum and CSF samples were collected at the same time, as described in more detail previously.^32^ However, plasma glucose was measured in samples collected at admission by the respective hospital laboratory. Briefly, CSF was collected at the induction of spinal anaesthesia prior to administering the anaesthetic agents. CSF samples were stored in polypropylene tubes at −80°C. Prior to storage, the samples were centrifuged and the supernatants aliquoted. Serum samples were obtained by venous puncture when CSF was sampled, and subsequently centrifuged, aliquoted and stored at −80°C. All samples were shipped on dry ice to the Bevital laboratory (www.bevital.no) for analyses of AcAc, β-HB, BCAAs and 3-HIB, using GC-MS/MS.^33^ Within- and between-day coefficients of variation ranged from 1.5% to 3.4%, and 1.8% to 4.0%, respectively. Technicians were blinded to clinical data. In a separate analysis, CSF lactate and glucose were measured with amperometry (Cobas b221).

### Statistics

The sample size was a convenience sample (i.e. all available CSF samples in an ongoing study). All metabolites showed positively skewed distributions, violating the assumption of normality in parametric analysis. Thus, univariate associations between metabolites and delirium were determined using the Mann–Whitney U-test. The association between normally distributed variables such as age and categorical variables such as sex and delirium were assessed using Student’s *t*-test or Pearson’s *χ*^2^, respectively. For the univariate analyses, we aimed to estimate an effect size that would be comparable for normal and non-normally distributed variables and categories. To achieve this aim, we normalized the area under the curve (AUC) obtained from the Mann–Whitney U-test or logistic regression so that zero represented no effect. The resulting Gini coefficient was calculated as [(2 × AUC) − 1].^34^ The subsequent scale [0 to 1 or 0 to −1 (i.e. positive or negative association)] represents effect sizes that can be approximated as weak (≥0.1 to <0.3), moderate (≥0.3 to <0.4) and strong (≥0.4).^35^

Next, we performed multivariable analyses using logistic regression with delirium as the outcome. Potential confounders were considered *a priori* and included age, sex, glomerular filtration rate (GFR), IQCODE (≥3.44 = dementia), ASA score (III-IV versus I-II) and diabetes. We also compared metabolite-metabolite correlations (Spearman's rho) and tested whether correlations were equivalent according to the presence of delirium using the Stata program CORTESTI.^36^ In supplementary analyses, we stratified the logistic regression according to the presence of dementia, which was not included as a potential confounder in this analysis. Furthermore, we assessed whether metabolite concentrations were more associated with delirium when it was subsyndromal, incident (occurred after surgery) or prevalent (already present at the time of surgery). Additional potential confounders were also included in supplementary analyses, namely: time from injury to admission, admission to surgery (which could indicate longer fasting) and injury to surgery, as well as body mass index (BMI) and plasma glucose. In cases of missing data, available cases were analysed. All analyses and figures were produced using StataCorp. 2023. Stata Statistical Software: Release 18. College Station, TX: StataCorp LLC.

## Results

### Study participants

Of the 406 patients included in the main analysis, 280 (69%) of the participants were female, 185 (46%) had dementia and 38 (9%) had diabetes (Table 1). In total, 224 (55%) of the participants were classified with delirium where 113 had delirium preoperatively and 108 developed delirium postoperatively (three participants could not be classified). Although the majority of patients with hip fracture were female, there was no predominance of gender for delirium. However, patients with delirium were 9 years older than those without delirium (mean 85 versus 76 years; *P* < 0.001). The delirium group had higher ASA score (68% versus 32% with ASA > II; *P* < 0.001), and more patients had dementia (IQCODE > 3.44) (71% versus 14%; *P* < 0.001). In addition, the delirium group had lower GFR compared with those without delirium (mean 67 versus 74 ml/min/1.73 m^2^; *P* > 0.001). The groups (delirium versus non-delirium) did not differ regarding BMI or diabetes. See the Supplementary material for associations between metabolites and the *a priori* selected confounders’ data for age, GFR, BMI, sex, dementia, comorbidity and diabetes (Supplementary Table 1).

**Table 1:** **Characteristics of patients with hip fracture according to delirium status (*n* = 406)** ^a^

Variable	No delirium	Delirium	GC^b^	*P*-value
	*n* = 182	*n* = 224		
**Demographics and clinical characteristics**				
Age^c^	75.8 ± 11.5	84.8 ± 7.5	0.48	<0.001**
Female^d^	130 (71)	150 (67)	0.04	0.686
IQCODE ≥ 3.44^d^, *n*(%)	25 (14)	160 (71)	0.58	<0.001**
ASA III-IV^d^	58 (32)	152 (68)	0.36	<0.001**
eGFR^c^	74.4 ± 19.2	66.6 ± 19.2	0.08	<0.001**
BMI^c,e^	23.6 ± 4.4	22.9 ± 3.5	0.08	0.102
Diabetes^d^	12 (7)	26 (12)	0.06	0.085

**CSF metabolite concentrations according to delirium status**				
Insulin resistance				
Isoleucine^f^	5.50 [2.96]	5.86 [3.45]	0.08	0.145
Leucine^f^	16.0 [6.77]	17.1 [7.07]	0.12	0.027*
Valine^f^	24.0 [9.88]	27.1 [11.8]	0.18	0.003*
3-HIB^f^	15.4 [4.31]	16.8 [5.36]	0.20	<0.001**
Energy metabolism				
β-HB^f^	31.3 [41.8]	48.2 [58.2]	0.20	<0.001**
Acetoacetate^f^	23.6 [26.4]	31.2 [38.4]	0.16	0.005*
Lactate^f,g^	1.85 [0.37]	1.96 [0.40]	0.30	<0.001**
Glucose^f,g^	4.37 [0.95]	4.44 [1.00]	0.04	0.493

3-HIB = 3-hydroxyisobutyrate; ASA = American Society of Anesthesiologists physical status classification; β-HB = β-hydroxybutyrate; BMI = body mass index; eGFR = glomerular filtration rate; GC = Gini coefficient; IQCODE = Informant Questionnaire on Cognitive Decline in the Elderly. All metabolites are in µmol/l, except lactate and glucose (mmol/l). Effect sizes calculated from logistic regression (clinical and demographic variables) or Mann–Whitney U-test (metabolites) with *P*-values from Student’s *t*-test (age, GFR, BMI), Pearson’s *χ*^2^ (female, IQCODE, ASA and diabetes) and Mann–Whitney U-test (metabolites).

^a^Forty-four patients with subsyndromal delirium were excluded.

^b^Gini coefficient [(2 × area under the curve) − 1]. The Gini coefficient here is comparable for all variables. The subsequent scale (0 to 1 or 0 to −1) represents effect sizes that can be considered weak (≥0.1 to <0.3), moderate (≥0.3 to <0.4) and strong (≥0.4). Statistical significance: *P < 0.05, **P < 0.001.

^c^Mean ± standard deviation.

^d^Number and (per cent) with trait according to delirium status.

^e^Missing data: BMI measured in 334 of 406 participants.

^f^Median and [interquartile range], i.e. the total distance between the 25th and 75th percentiles.

^g^Missing data: 307 of 406 had measured glucose and lactate.

### Metabolites and delirium

#### Univariate analyses

**Figure 1:**
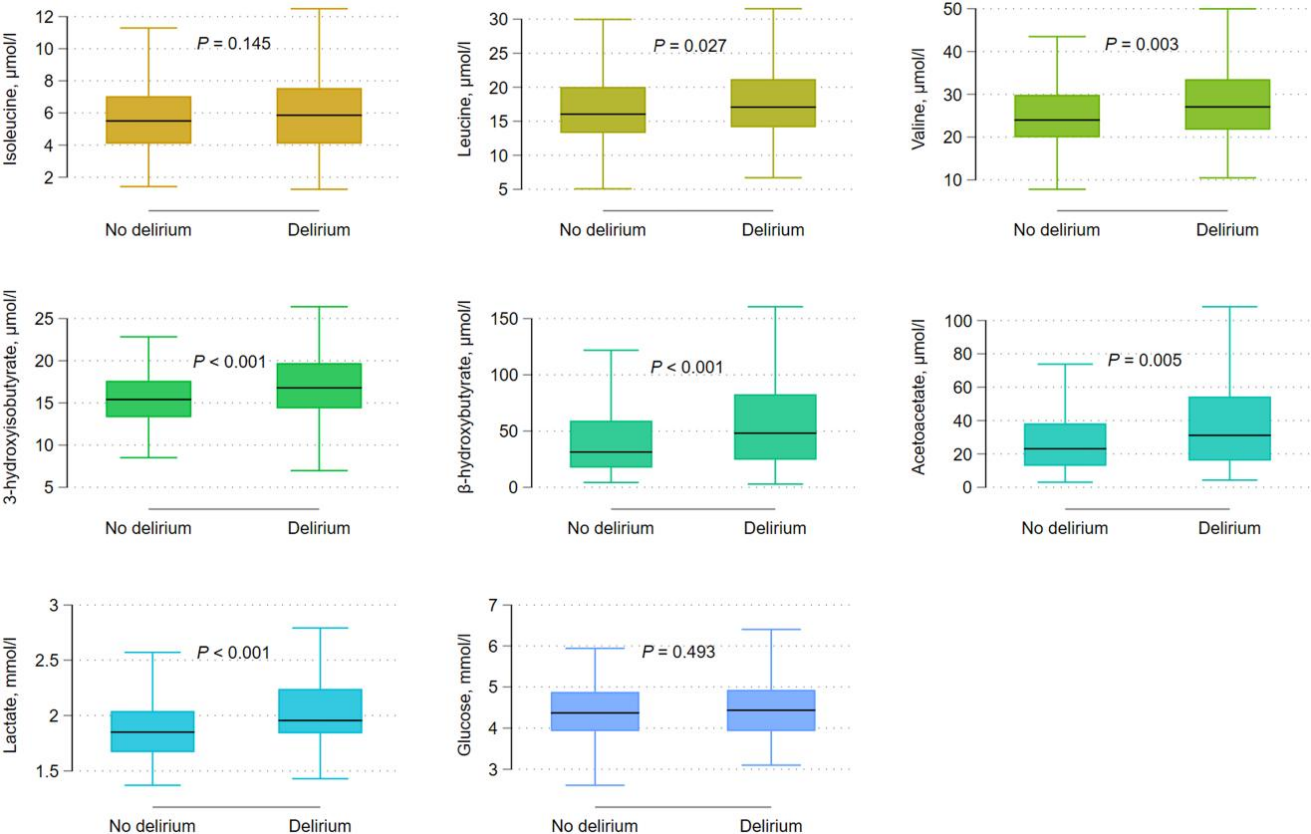
**Box and whisker plots of CSF metabolites according to delirium status.** The line in the middle of the boxes indicates the median, the upper and lower edges of the boxes indicate the 25th and 75th percentiles (lower and upper quartiles). The whiskers indicate the most extreme values within the 1.5 interquartile range of the upper or lower quartile; *n* = 406.

CSF concentrations of the BCAA leucine and, particularly, valine and its catabolite 3-HIB were associated with delirium, as were the CSF concentrations of the ketone bodies β-HB and AcAc. Lactate (only measured in CSF) was the metabolite most strongly associated with delirium (Table 1 and Fig. 1). In the sub-cohort with paired serum and CSF samples (Table 2), BCAAs and ketone bodies were associated with delirium in the CSF only. 3-HIB was the only metabolite associated with delirium in both serum and CSF. Glucose was not associated with delirium in either serum or CSF.

**Table 2:** Metabolites by delirium in a subgroup with paired serum and CSF samples (*n* = 213)

	Serum^a^				CSF^a^			
	Delirium		Group difference		Delirium		Group difference	
Mtb	No	Yes	GC^b^	*P*	No	Yes	GC^b^	*P*
Ile	52.8 [20]	57.3 [29]	0	0.968	5.78 [2.9]	6.35 [2.9]	0.14	0.072
Leu	123 [43]	129 [44]	0.04	0.632	16.8 [6.6]	17.8 [6.8]	0.18	0.025*
Val	225 [57]	232 [58]	0.04	0.567	24.8 [9.3]	28.4 [13]	0.20	0.009*
3-HIB	13.0 [6.7]	14.8 [6.3]	0.24	<0.001**	15.4 [4.3]	16.9 [5.0]	0.26	0.001*
β-HB	264 [386]	316 [462]	0.06	0.486	31.6 [42]	49.9 [59]	0.24	0.002*
AcAc	117 [186]	126 [194]	0.06	0.485	22.4 [23]	31.1 [38]	0.18	0.019*
Lac^c^					1.85 [0.4]	1.95 [0.4]	0.30	<0.001**
Glu^c,d^	6.20 [1.6]	6.50 [2.1]	0.06	0.334	4.37 [1.0]	4.33 [0.9]	−0.02	0.851

3-HIB = 3-hydroxyisobutyrate; AcAc = acetoacetate; β-HB = β-hydroxybutyrate; Glu = glucose; Ile = isoleucine; Lac = lactate; Leu = leucine; Mtb = metabolite. *P < 0.05, **P < 0.001.

^a^All metabolites in median and [interquartile range], with interquartile range as the distance between the 25th and 75th percentiles.

^b^Gini coefficient [(2 × area under the curve) − 1]. The Gini coefficient here is comparable for all variables. The subsequent scale (−1 to 1) represents effect sizes that can be considered weak (≥0.1 to <0.3), moderate (≥0.3 to <0.4) and strong (≥0.4).

^c^CSF glucose and lactate (lactate only in CSF) measured in 199 participants with serum samples (glucose measured in plasma).

^d^Plasma glucose measured at admission (all other at the time of induction of spinal anaesthesia) in 304 participants.

#### Multivariable analysis

**Figure 2:**
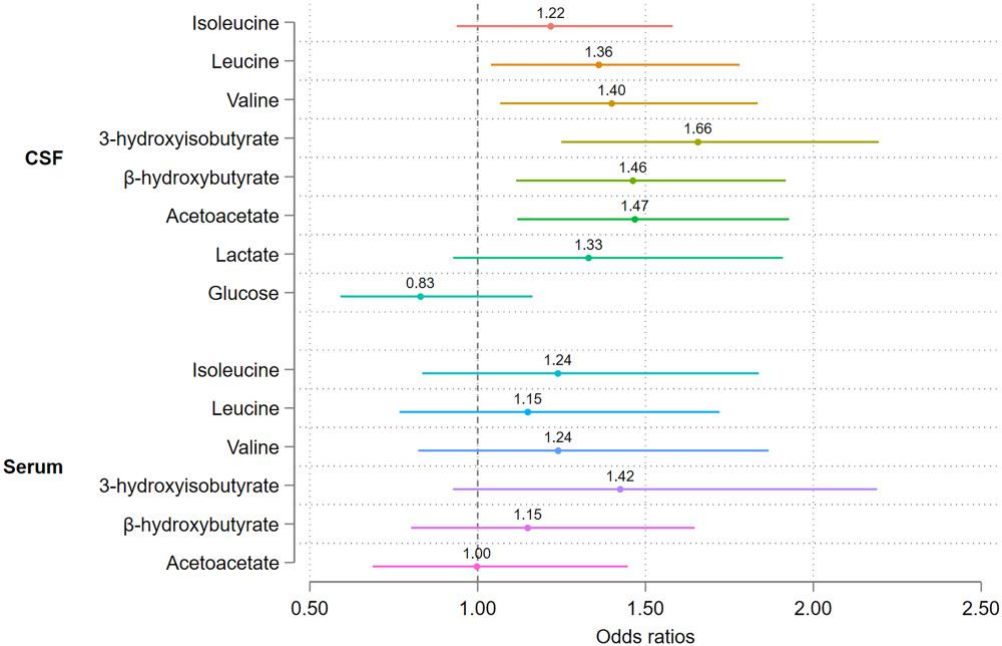
**Metabolite concentrations and the odds of delirium.** Logistic regression with delirium as the outcome adjusted for age, sex, cognitive impairment (IQCODE ≥3.44), American Society of Anesthesiologists (ASA) score (III-IV versus I-II), glomerular filtration rate (eGFR) and diabetes. The 95% confidence intervals that do not cross the vertical dashed line at 1.00 represent statistically significant findings. The analysis contains serum samples (except for glucose, which was measured in plasma) (*n* = 213) and CSF samples (*n* = 406).

Using logistic regression adjusted for age, sex, GFR, BMI, dementia, ASA and diabetes, CSF 3-HIB was most strongly associated with delirium among the measured metabolites. CSF concentrations of AcAc, β-HB, leucine and valine all remained significantly associated with delirium after adjustment for covariates. Lactate, however, was confounded in the adjusted model. No serum metabolites were significantly associated with delirium, and notwithstanding their lower sample size, also had weaker effect sizes (Fig. 2).

Additional adjustments for BMI, time from injury to admission, time from admission to surgery and time from injury to surgery did not significantly affect the results (Supplementary Tables 2–4). See Supplementary Fig. 1 for analysis stratified by the presence of dementia. Furthermore, we evaluated the association between CSF metabolite concentrations and delirium according to its clinical classification (prevalent versus incident versus subsyndromal delirium). Briefly, ketone bodies in the CSF were more strongly associated with delirium in patients without dementia (Supplementary Fig. 1) and in patients who had delirium at the time of CSF sampling (prevalent) rather than in patients who developed delirium later (incident; Supplementary Fig. 2).

#### Metabolite-metabolite correlations according to delirium

Assessing all possible correlations between the measured metabolites, we found that in patients with delirium, there was a stronger negative Spearman correlation between CSF glucose and both CSF and serum ketone bodies (Table 3), and that CSF lactate was less dependent on CSF glucose in patients with delirium. We did not include plasma glucose as it was measured on admission. The pairwise CSF:serum correlations for ketone bodies, BCAAs and 3-HIB did not differ significantly between the groups.

**Table 3:** Metabolite-metabolite correlations in hip fracture patients by delirium status

		CSF								Serum					
		Ile	Leu	Val	3-HIB	β-Hb	AcAc	Lac	Glu	Ile	Leu	Val	3-HIB	β-Hb	AcAc
**CSF**	**Ile**	1	–	–	–	–	–	–	–	–	–	–	–	–	–
		**1**	–	–	–	–	–	–	–	–	–	–	–	–	–
	**Leu**	0.84	1	–	–	–	–	–	–	–	–	–	–	–	–
		**0**.**82**	**1**	–	–	–	–	–	–	–	–	–	–	–	–
	**Val**	0.77	0.92	1	–	–	–	–	–	–	–	–	–	–	–
		**0**.**76**	**0**.**91**	**1**	–	–	–	–	–	–	–	–	–	–	–
	**3-HIB**	0.24	0.39	0.40	1	–	–	–	–	–	–	–	–	–	–
		**0**.**30**	**0**.**41**	**0**.**42**	**1**	–	–	–	–	–	–	–	–	–	–
	**β-Hb**	0.27	0.37	0.27	0.52	1	–	–	–	–	–	–	–	–	–
		**0**.**35**	**0**.**41**	**0**.**27**	**0**.**51**	**1**	–	–	–	–	–	–	–	–	–
	**AcAc**	0.22	0.35	0.23	0.51	0.95	1	–	–	–	–	–	–	–	–
		**0**.**30**	**0**.**36**	**0**.**19**	**0**.**42**	**0**.**95**	**1**	–	–	–	–	–	–	–	–
	**Lac**	0.31	0.30	0.35	0.32	0.20	0.10	1	–	–	–	–	–	–	–
		**0**.**23**	**0**.**20**	**0**.**23**	**0**.**25**	**0**.**16**	**0**.**08**	**1**	–	–	–	–	–	–	–
	**Glu**	−0.02	−0.03	0.07	0.09	−0.09^a^	−0.13^b^	0.45^c^	1	–	–	–	–	–	–
		**−0**.**07**	**−0**.**10**	**−0**.**01**	**−0**.**05**	**−0**.**32**^a^	**−0**.**36**^b^	**0.21** ^c^	**1**	–	–	–	–	–	–
**Serum**	**Ile**	0.51	0.41	0.27	0.26	0.41	0.45	0.05	−0.19	1	–	–	–	–	–
		**0**.**56**	**0**.**46**	**0**.**25**	**0**.**31**	**0**.**42**	**0**.**42**	**−0.01**	**−0.22**	**1**	–	–	–	–	–
	**Leu**	0.27	0.46	0.30	0.40	0.54	0.59	0.06	−0.19	0.78	1	–	–	–	–
		**0**.**30**	**0**.**48**	**0**.**25**	**0**.**35**	**0**.**51**	**0**.**52**	**−0.06**	**−0.29**	**0.82**	**1**			–	–
	**Val**	0.26	0.44	0.37	0.47	0.40	0.45	0.04	−0.17	0.71	0.91	1	–	–	–
		**0**.**32**	**0**.**53**	**0**.**39**	**0**.**43**	**0**.**49**	**0**.**50**	**−0.04**	**−0.25**	**0.72**	**0.91**	**1**	–	–	–
	**3-HIB**	0.27	0.43	0.32	0.51	0.37	0.35	0.26	0.18^d^	0.48	0.58	0.58	1	–	–
		**0**.**33**	**0**.**38**	**0**.**23**	**0**.**54**	**0**.**34**	**0**.**28**	**0.35**	**−0.12** ^d^	**0.43**	**0.45**	**0.39**	**1**	–	–
	**β-Hb**	0.11	0.27	0.12	0.30	0.83	0.84	0.03	−0.24	0.46	0.58	0.40	0.35	1	–
		**0**.**18**	**0**.**26**	**0**.**10**	**0**.**31**	**0**.**79**	**0**.**79**	**0.01**	**−0.46**	**0.47**	**0.56**	**0.49**	**0.37**	**1**	–
	**AcAc**	0.14	0.24	0.14	0.20	0.67	0.73	0.04	−0.14^e^	0.36	0.43	0.27	0.18	0.81	1
		**0**.**16**	**0**.**25**	**0**.**09**	**0**.**24**	**0**.**65**	**0**.**71**	**−0.09**	**−0.41** ^e^	**0.44**	**0.51**	**0.46**	**0.27**	**0.82**	**1**

Correlogram of Spearman correlation coefficients in patients without (not bold) or with (in bold) delirium. CSF metabolites measured in 406 patients except glucose and lactate (*n* = 307) and serum metabolites measured in 213 patients (glucose measured in plasma). 3-HIB = 3-hydroxyisobutyrate; AcAc = acetoacetate; β-HB = β-hydroxybutyrate; Glu = glucose (CSF only); Ile = isoleucine; Lac = lactate (CSF only); Leu = leucine; Val = valine.

^a-e^Metabolite-metabolite correlations that were significantly different between the groups.

## Discussion

To our knowledge, this is the first study to examine metabolic biomarkers linked to insulin resistance and ketone body metabolism in the CSF of patients with delirium. We demonstrate elevated CSF concentrations of BCAAs (leucine and valine), 3-HIB (a valine metabolite), and the ketone bodies, AcAc and β-HB, in patients with delirium. Notably, these changes were observed despite no difference in serum and CSF glucose concentrations, and the identified metabolic alterations were mostly absent in the blood. Based on the data in this study, we propose that impaired glucose utilization occurs in the brain during delirium, with brain insulin resistance being a possible mechanism.

Our findings of no difference in blood glucose concentrations at hospital admission and CSF glucose concentration peroperatively between the delirium and no-delirium groups are similar to results from previous studies in smaller cohorts.^6,10^ We found that lactate was the most elevated metabolite in delirium using univariate analysis, in line with previous findings.^6,10^ However, the effect size was attenuated in adjusted analyses, with the lactate-delirium association could be largely explained by lactates link with age, comorbidity, cognitive impairment and diabetes (Supplementary Table 1). Differences in control groups and age composition make it difficult to compare our adjusted analyses with results from previous reports.^6^

Despite no differences in blood and CSF glucose concentrations, we found higher CSF levels of the BCAA’s leucine, valine and the valine catabolite 3-HIB in the delirium group, even after adjustment in multivariable analysis. Among these metabolites, only 3-HIB was also elevated in serum. Increased circulating BCAAs and 3-HIB are strongly linked with insulin resistance^21,22,37^ but have not previously been validated for this purpose in human CSF. Significantly, we also observed elevation of the BCAAs and 3-HIB in both the serum and CSF of patients with diabetes, suggesting that insulin resistance also affects CSF concentrations. Whether the BCAAs contribute to a state of insulin resistance or rather accumulate due to a lack of insulin is a matter of debate.^19,38^ 3-HIB, which is strongly associated with diabetes mellitus type 2 and insulin resistance,^21^ was the only metabolite significantly associated with delirium both in blood and CSF. Neurons catabolize valine for use in energy metabolism, and 3-HIB may negatively affect energy metabolism in neurons.^39^

Higher CSF concentrations of the ketone bodies, β-HB and AcAc, were associated with delirium in univariate and adjusted analyses. Again, this was not observed in serum. The liver produces ketone bodies, which are used peripherally and by the brain as an energy source during situations of glucose shortage, such as fasting or prolonged exercise, or in pathological conditions such as insulin resistance.^40^ CSF concentrations are believed to reflect mainly peripheral concentrations^23^ and indeed CSF:serum correlations of ketone bodies were strong in the present study (Table 3). However, the elevated CSF concentrations of ketone bodies in delirium cannot be fully explained by serum concentrations, which were similar in patients with and without delirium, as was CSF:serum correlations for ketones and other metabolites. It has been shown that astrocytes may oxidize fatty acids to produce ketones that can be used as an energy source.^24^ Thus, our findings might be explained by increased production of ketone bodies in the brain, increased uptake from the blood or by decreased ketone utilization by the brain during delirium. These possibilities require further research.

The CSF metabolite profile of patients with hip fracture suggests, intriguingly, an increase in both aerobic fatty acid oxidation (ketones) and anaerobic glycolysis (lactate) in patients with delirium. According to differences in correlation, the ketones were more inversely associated with glucose in the CSF of patients with delirium, suggesting they may be more readily generated in delirium. Furthermore, CSF lactate seems to be produced by processes that are less related to CSF glucose concentrations in patients with delirium compared to those without (Table 3). Such uncoupling could, for example, be seen with disturbed microcirculation during acute illness when blood containing glucose is not distributed optimally or equally to all cells.^41^ This may result in significant alterations in energy homeostasis and utilization of glucose in the brain during delirium. Potential explanations of our findings include brain insulin deficiency or brain insulin resistance, which would account for increased leucine, valine and 3-HIB, biomarkers strongly associated with insulin resistance, as well as increased ketone body formation. Activation of AMP-activated protein kinase (AMPK), a key sensor of brain energy balance,^42^ could partly account for increased ketogenesis in astrocytes and a switch to anaerobic glycolysis and breakdown of amino acids. This could be related to ischaemia, hypoxia or fasting, although the latter should also have given rise to changes in circulating concentrations. Importantly, our study cannot determine whether the observed alterations in brain metabolism are in themselves harmful or whether they reflect an adaptive response to cellular energy deficiency.

Our study has several strengths, most importantly a relatively large sample size with available CSF and a comprehensive prospective evaluation for delirium. We used a targeted metabolomics approach in order to evaluate two specific pathways, with the advantage of metabolites measurement with high-precision and unequivocal identification, but with limited coverage. Future studies should seek to more comprehensively map metabolic pathways of energy metabolism. From a clinical perspective, it should be investigated whether these CSF alterations have external validity in other populations at risk of delirium such as those with infectious diseases.

We lack precise information regarding the fasting time, from the last meal to assessment of delirium, as patients would typically be put on a preoperative fasting schedule only to be interrupted in case of rescheduling of the surgery. Still, the total time from injury/admission to surgery should, to some extent, adjust for fasting (Supplementary Table 2). Moreover, plasma glucose was measured at admission, while CSF samples, including glucose, were taken at the time of spinal anaesthesia [median 23 h from admission to surgery in patients with hip fracture without delirium and 25 h with delirium (the difference was not significant); Supplementary Table 2]. In addition, given the clear changes in multiple metabolites, it would be of interest to pursue repeated serum and CSF sampling in order to examine trajectories of metabolic changes. Although the latter would be difficult to accomplish in practice, it might be achieved in smaller studies. Fertleman *et al*.^43^ identified changes in CSF cytokine levels in surgical patients with a fractured femoral neck by collecting CSF samples through a spinal catheter inserted before surgery.

Another constraint in this study was related to the assessment of pre-fracture dementia status using the IQCODE, a validated and frequently used assessment tool in studies of acutely admitted patients.^44^ However, it is not a substitute for a complete dementia assessment with objective cognitive testing. Some patients (*n* = 30) had missing data on the IQCODE. In such cases, the dementia status was decided retrospectively, based on information in the hospital records case notes, and could have introduced diversity into the dataset.

An insufficiency in cerebral energy metabolism has been proposed as a causal hypothesis of delirium for more than 60 years, supported by studies of experimental hypoxia and hypoglycaemia and by findings of glucose hypometabolism in PET studies. Our study suggests significant local changes in brain energy metabolism occur in patients with delirium. The underlying mechanisms explaining increased levels of ketone bodies, lactate and BCAAs in CSF in combination with no change in peripheral concentrations of these metabolites are unclear, but potentially linked to brain insulin resistance. Our data highlight the value of studies utilizing CSF in the investigation of delirium.

## Supplementary Material

awad296_Supplementary_Data

## Data Availability

Owing to ethical restrictions, the full dataset is available to the reader upon request only. Proposals should be directed to the corresponding author at Giil.Melver@uib.no and to gain access, data requestors will need to sign a data access agreement.
