# Supplementary material for: Impaired glucose utilization in the brain of patients with delirium following hip fracture
Source: Brain. 2023 Sep 2;147(1):215–23. doi: 10.1093/brain/awad296 (PMC10766236; doi:10.1093/brain/awad296)
Supplement: awad296_Supplementary_Data [file awad296_supplementary_data.zip › brain-2022-02257-File007.pdf]

# Supplementary material

## Supplementary methods

### Definition of subsyndromal delirium

In the first cohort, subsyndromal delirium was defined when the patients fulfilled a minimum of two elements of the Confusion Assessment Method (CAM) without fulfilling the diagnostic criteria of delirium.[1] In the second cohort, subsyndromal delirium was defined when not all the Diagnostic and Statistical Manual of Mental Disorders 5 (DSM-5) criteria were completely fulfilled. However, the patients had acutely altered cognition, including any of the following: (a) altered arousal, (b) impaired attention, (c) additional cognitive alterations, (d) change in perception.[2]

## Supplementary results

### Metabolites and potential confounders

The aim of the supplementary analyses was to assess the associations between metabolites and potential confounders and delirium, including confounders that were not considered as confounders a priori were worth exploring. We included measures where we did not have complete data for subanalysis of the patients where these data were measured. Hence, univariate analyses are run on complete cases. Further, all multivariable analyses include the confounders from the main analysis (age, gender, GFR, IQCODE, ASA score and diabetes) and then one separate analysis was run per additional confounder with missing data. We assessed associations between metabolites and continuous variables (age, BMI, GFR) using Spearman Rho's correlation coefficients. For categorical variables, we used the Mann-Whitney U test, where we calculated an effect size from the area under the curve (AUC) which is equivalent to the Mann-Whitney U effect size. We calculated the Gini coefficient based on the area under the curve (2*AUC-1). Whereas the AUC has the neutral point at 0.5 and no negative values (range zero to one), the Gini Coefficient has no effect at zero and has a range from minus one to plus one. Briefly, BCAA, 3-HIB, lactate, and glucose were higher in patients with diabetes mellitus in both serum and CSF. Several associations between the potential confounders, age,

eGFR, BMI, sex, ASA score, and metabolites are listed in Supplementary Table 1, where CSF lactate and serum 3-HIB displayed significant associations with most confounders.

**Supplementary Table 1 Cerebrospinal fluid (*N* = 406) and serum (*N* = 213) metabolites correlations and covariates**

| | **Continuous variables** *Spearman correlations* | | | **Categorical variables** *Gini coefficients*[a] | | | |
|---|---|---|---|---|---|---|---|
| **CSF** | **Age** | **GFR** | **BMI**[b] | **Female** | **IQCODE** | **ASA** | **Diabetes** |
| Isoleucine | 0.03 | -0.09* | 0.09 | -0.22** | -0.06 | 0.12* | 0.26* |
| Leucine | 0.01 | -0.08 | 0.13* | -0.26** | -0.02 | 0.12* | 0.32** |
| Valine | 0.09 | -0.11* | 0.15* | -0.22** | -0.02 | 0.14* | 0.34** |
| 3-HIB | 0.08 | -0.13* | 0.07 | -0.26** | 0.02 | 0.08 | 0.30** |
| β-HB | 0.08 | 0.06 | -0.10* | -0.14* | 0.10 | 0.12* | -0.06 |
| AcAc | 0.04 | 0.06 | -0.08 | -0.10 | 0.08 | 0.08 | -0.12 |
| Lactate[c] | 0.31** | -0.12* | 0.03 | -0.20* | 0.22** | 0.28** | 0.28* |
| Glucose[c] | 0.12* | -0.15* | 0.13* | 0 | 0.14* | 0.12 | 0.64** |
| **Serum** | | | | | | | |
| Isoleucine | -0.14* | -0.07 | 0.04 | -0.12 | -0.08 | 0.08 | 0.20 |
| Leucine | -0.11 | -0.07 | 0.05 | -0.20* | 0.04 | 0.02 | 0.19 |
| Valine | -0.12 | -0.08 | 0.19** | -0.18* | 0 | 0.02 | 0.42** |
| 3-HIB | 0.13* | -0.29** | 0.02 | -0.42** | 0.16* | 0.22* | 0.50** |
| β-HB | -0.02 | 0.02 | -0.21** | -0.16* | 0.06 | 0.06 | -0.18 |
| AcAc | 0.02 | -0.07 | -0.13 | -0.08 | 0.08 | 0.08 | -0.10 |
| Glucose[d] | 0.23** | -0.15* | 0.02 | 0.08 | 0.10 | 0.16* | 0.62** |

Abbreviations: 3-HIB, 3-hydroxyisobutyrate; AcAc, acetoacetate; ASA, American Society of Anesthesiologists (ASA) physical status classification; β-HB, β-hydroxybutyrate; BMI, Body mass index; eGFR, glomerular filtration rate; IQCODE, Informant Questionnaire on Cognitive Decline in the Elderly.

[a] Gini coefficient ((2*Area under the curve)-1). The Gini coefficient is here comparable for all variables. The subsequent scale (-1 to 1) represents effect sizes that can be considered weak (≥ 0.1 to <0.3), moderate (≥ 0.3 to <0.4), and strong (≥ 0.4).

[b] *N* = 332 with BMI measured and CSF metabolites, *N* = 170 with BMI measured and serum metabolites (glucose measured in plasma)

[c] *N* = 307 measurements

[d] *N* = 304 measurements (measured in routine clinical plasma samples)

IQCODE ≥3.44 denotes cognitive impairment

ASA score dichotomized (I-II = 0, III-IV = 1)

* *P*-value < 0.05, ** *P*-value < 0.001

# Metabolites and waiting times

The number of hours from injury to admission, from admission to surgery, and from injury to surgery could clearly affect both the risk of delirium and energy metabolism by way of fasting.[3,4] These times were only marginally higher in patients with delirium but far from significant (Supplementary Table 2). The correlation between admission to surgery and injury to surgery with BCAAs and ketone bodies was strongest for serum concentrations, although several were also significant in the CSF. There was a negative correlation between the time from injury or admission to surgery and CSF glucose and lactate (Supplementary Table 3). As there were no differences in these time measurements (Supplementary Table 2) by delirium, it was not surprising that these times did not attenuate associations between metabolites and delirium. Furthermore, plasma glucose on admission was also not attenuate the associations

between metabolites and delirium and neither did body-mass index, the latter considering more long-term alterations (Supplementary Table 4).

**Supplementary Table 2 Time intervals in hours for hip fracture patients**

| Hours from | *N*[a] | No delirium[b] | Delirium[b] | GC[c] | *P*-value[d] |
|---|---|---|---|---|---|
| Injury to admission | 354 (199 vs 155) | 2.0 ±5 | 2.8 ±6 | 0.08 | 0.181 |
| Admission to surgery | 400 (220 vs 180) | 23 ±18 | 25 ±22 | 0.04 | 0.407 |
| Injury to surgery | 350 (196 vs 154) | 28 ±24 | 29 ±25 | 0.02 | 0.778 |

[a] Number with available data out of 406 (*N* without delirium vs *N* with delirium)

[b] Values in median ±interquartile range

[c] Gini coefficient ((2*Area under the curve)-1). The Gini coefficient is here comparable for all variables. The subsequent scale (-1 to 1) represents effect sizes that can be considered weak (≥ 0.1 to <0.3), moderate (≥ 0.3 to <0.4), and strong (≥ 0.4).

[d] Mann-Whitney U test

**Supplementary Table 3 Spearman correlations between metabolites and time intervals**

| | **Time from:** | | |
|---|---|---|---|
| **CSF** | **Injury to admission** (*n* = 354) | **Admission to surgery** (*n* = 400) | **Injury to surgery** (*n* = 350) |
| Isoleucine | -0.01 | 0.03 | 0.04 |
| Leucine | 0.05 | 0.12* | 0.15* |
| Valine | 0.04 | 0.06 | 0.08 |
| 3-hydroxyisobutyrate | 0.08 | 0.10* | 0.12* |
| β-hydroxybutyrate | 0.08 | 0.10* | 0.12* |
| Acetoacetate | 0.11* | 0.12* | 0.16* |
| | (*n* = 258) | (*n* = 303) | (*n* = 255) |
| Lactate | -0.02 | -0.10 | -0.14* |
| Glucose | -0.09 | -0.13* | -0.18* |
| **Serum** | (*n* = 198) | (*n* = 235) | (*n* = 196) |
| Isoleucine | 0.01 | 0.27* | 0.28* |
| Leucine | 0.05 | 0.25* | 0.32* |
| Valine | 0.05 | 0.27* | 0.32* |
| 3-hydroxyisobutyrate | 0.06 | 0.17* | 0.21* |
| β-hydroxybutyrate | 0.04 | 0.12 | 0.15* |
| Acetoacetate | 0.03 | 0.17* | 0.19* |

* *P*-value < 0.05

**Supplementary Table 4 Additional adjustment for potential confounders that also had missing data[a]**

| CSF metabolites | OR | CI | *P*-value |
|---|---|---|---|
| | Additional adjustment for body mass index (*N* = 332) | | |
| Leucine | 1.35 | 1.01, 1.82 | 0.044* |
| Valine | 1.37 | 1.02, 1.83 | 0.037* |
| 3-HIB | 1.72 | 1.27, 2.35 | 0.001* |
| β-Hb | 1.41 | 1.06, 1.87 | 0.019* |
| AcAc | 1.44 | 1.08, 1.92 | 0.011* |
| | Additional adjustment for the time from admission to surgery (*n* = 398) | | |
| Leucine | 1.33 | 1.01, 1.75 | 0.041* |
| Valine | 1.38 | 1.05, 1.81 | 0.023* |
| 3-HIB | 1.64 | 1.24, 2.19 | 0.001* |
| β-Hb | 1.44 | 1.10, 1.89 | 0.009* |
| AcAc | 1.46 | 1.10, 1.90 | 0.009* |
| | Additional adjustment for the time from injury to admission (*n* = 351) | | |
| Leucine | 1.23 | 0.93, 1.64 | 0.145 |
| Valine | 1.29 | 0.97, 1.72 | 0.077 |
| 3-HIB | 1.63 | 1.20, 2.20 | 0.002* |
| β-Hb | 1.34 | 1.01, 1.78 | 0.045* |
| AcAc | 1.38 | 1.03, 1.85 | 0.029* |
| | Additional adjustment for the time from injury to surgery (*n* = 348) | | |
| Leucine | 1.26 | 0.94, 1.67 | 0.116 |
| Valine | 1.32 | 0.99, 1.75 | 0.058 |
| 3-HIB | 1.63 | 1.21, 2.12 | 0.001* |
| β-Hb | 1.36 | 1.03, 1.81 | 0.033* |
| AcAc | 1.40 | 1.05, 1.88 | 0.021* |
| | Additional adjustment for serum glucose (*n* = 302) | | |
| Leucine | 1.40 | 1.03, 1.90 | 0.034* |
| Valine | 1.44 | 1.06, 1.98 | 0.022* |
| 3-HIB | 1.66 | 1.19, 2.30 | 0.003* |
| β-Hb | 1.49 | 1.09, 2.03 | 0.013* |
| AcAc | 1.49 | 1.08, 2.06 | 0.014* |

Abbreviations: 3-HIB, 3-hydroxyisobutyrate; AcAc, acetoacetate; β-Hb, β-hydroxybutyrate; CI, 95% confidence interval; OR, odds ratio.

[a] Logistic regression with delirium as the outcome and age, sex, ASA score (III-IV vs I-II), IQCODE (≥3.44), estimated glomerular filtration rate, diabetes as covariates and the listed additional covariates and metabolites. Cases with missing values were excluded from the analysis. This was done separately for each potential confounder.

**P*-value < 0.05

# Metabolites and delirium by subgroups

## By dementia

In the stratified logistic regression adjusted for covariates (see Statistics in the main text) associations between CSF 3-HIB, β-HB, and AcAc and delirium were observed mainly in patients without dementia (Supplementary Fig. 1). Although not significant in either subgroup, lactate was more strongly associated with delirium in patients without dementia, whereas the BCAA's were more associated with delirium in patients with dementia.

## By clinical classification of delirium

Here, we additionally examined the univariate associations of metabolites with delirium according to whether delirium occurred prior to CSF sampling (prevalent delirium) or arose

postoperatively (incident delirium) (Supplementary Fig. 2). Of the BCAAs, valine, and its catabolite 3-HIB were significantly associated with incident and prevalent delirium compared to no delirium. Ketone bodies were most strongly associated with prevalent delirium and lactate was more strongly associated with incident delirium.

## Supplementary discussion

Longer duration of surgery has been associated with an increased risk of developing postoperative delirium.[5,6] We found that a longer waiting time for surgery was associated with an increase in BCAAs and ketone bodies in serum and CSF but lower CSF lactate and glucose concentration (correlation with plasma glucose could not be calculated since this was measured only at admission). This suggests that these patients were indeed experiencing the metabolic effects of fasting (Supplementary Table 3). Patients with delirium had longer waiting time for surgery compared to those without (25 versus 23 hours, $P = 0.407$), see Supplementary Table 2. Moreover, we adjusted the analysis for *waiting time* since this has been identified as a risk factor for delirium in previous studies. However, we found no indication that waiting time confounded our findings (Supplementary Table 4). There was also no confounding regarding plasma glucose at admission and BMI. This further underscores the likelihood that changes in brain energy metabolism are afoot in delirium, reflected by CSF alterations and not systemic processes (Supplementary Tables 1 and 4). We did not measure the duration of surgery but it bears no relevance at CSF metabolites that were collected at the induction of spinal anesthesia.

The ketone bodies, AcAc and β-HB, were found to be more strongly associated with prevalent compared to incident delirium. In contrast, 3-HIB had the strongest association with subsyndromal delirium although valine, the precursor amino acid, was not (Supplementary Fig. 2). This strengthens our presumption that the clinical classification of delirium (prevalent, incident, or subsyndromal) may determine the concentrations of the metabolites. Yet, it may also raise a question regarding the interpretation of the results, as the differences across groups point to the direction of *markers of delirium* (prevalent delirium), while changes in the group yet to develop delirium (incident delirium) are more related to *risk factors* for delirium. Our study cannot inform of potential alterations in the concentrations of the metabolites (from non-delirium to delirium or from delirium to non-delirium state) as CSF samples were collected only once. However, the findings indicate an association between delirium and elevated concentrations of 3-HIB and ketones, regardless of whether the patients developed pre- or postoperative delirium.

Previous evidence suggests that insulin resistance is a central contributor to the pathogenesis of neurodegenerative cognitive diseases such as dementia.[7] However, in our study, there was a weaker association between 3-HIB concentrations and delirium among patients with dementia, although the associations with BCAAs were somewhat stronger. In general, patients with

neurodegenerative disease may have higher baseline levels of insulin resistance, perhaps offsetting any additional acute changes. Further, they have a much lower threshold of developing delirium, and thus other pathophysiological triggers may be present in dementia.

The ketone bodies, AcAc and β-HB, were more strongly associated with delirium among patients without dementia. CSF and serum AcAc were not correlated with dementia, or any potential confounders used in this study. However, CSF β-HB shows a weak though significant association with dementia (Supplementary, Table 1). Patients with dementia have a different threshold for developing delirium, and indeed pathophysiological pathways to delirium may differ among patients with and without neurodegenerative disease. From a methodological perspective, it can be difficult to both diagnose and rule out delirium superimposed on dementia making this group prone to misclassification bias with respect to delirium. Most patients with dementia in this cohort developed delirium, and thus, there are few patients with dementia and no delirium. Thus, the clinical uncertainty between delirium and no delirium is less in the group without dementia.

# Supplementary references


1. Halaas NB, Blennow K, Idland AV, et al. Neurofilament Light in Serum and Cerebrospinal Fluid of Hip Fracture Patients with Delirium. *Dement Geriatr Cogn Disord*. 2018;46(5-6):346-357. doi:10.1159/000494754

2. Pollmann CT, Mellingsæter MR, Neerland BE, Straume-Næsheim T, Årøen A, Watne LO. Orthogeriatric co-management reduces incidence of delirium in hip fracture patients. *Osteoporos Int*. Nov 2021;32(11):2225-2233. doi:10.1007/s00198-021-05974-8

3. Jin Z, Hu J, Ma D. Postoperative delirium: perioperative assessment, risk reduction, and management. *British Journal of Anaesthesia*. 2020;125(4):492-504. doi:https://doi.org/10.1016/j.bja.2020.06.063

4. Kong H, Xu LM, Wang DX. Perioperative neurocognitive disorders: A narrative review focusing on diagnosis, prevention, and treatment. *CNS Neurosci Ther*. Aug 2022;28(8):1147-1167. doi:10.1111/cns.13873

5. Lin Y, Chen J, Wang Z. Meta-Analysis of Factors Which Influence Delirium Following Cardiac Surgery. *Journal of Cardiac Surgery*. 2012;27(4):481-492. doi:https://doi.org/10.1111/j.1540-8191.2012.01472.x

6. Ravi B, Pincus D, Choi S, Jenkinson R, Wasserstein DN, Redelmeier DA. Association of Duration of Surgery With Postoperative Delirium Among Patients Receiving Hip Fracture Repair. *JAMA Network Open*. 2019;2(2):e190111-e190111. doi:10.1001/jamanetworkopen.2019.0111

7. Al Haj Ahmad RM, Al-Domi HA. Thinking about brain insulin resistance. *Diabetes & Metabolic Syndrome: Clinical Research & Reviews*. Nov 2018;12(6):1091-1094. doi:10.1016/j.dsx.2018.05.003

# Supplementary Figure legends

**Supplementary Figure 1. Cerebrospinal fluid metabolite concentrations and delirium according to dementia (IQCODE cutoff 3.44) at baseline (N = 221 without dementia, N = 185 with dementia)**. Logistic regression with delirium as the outcome, adjusted for age, sex, ASA score (III-IV vs I-II), glomerular filtration rate (eGFR) and diabetes. 95% confidence intervals who do not cross the vertical stapled line at 1.00 represent statistically significant findings.

**Supplementary Figure 2. Univariate association between cerebrospinal fluid metabolites and delirium according to clinical classification of delirium.** *Subsyndromal* represent participants ($n = 44$) who had multiple clinical findings suggestive of delirium but did not fulfill all diagnostic criteria (excluded from main analyses), *incident* represent patients who developed delirium postoperatively, and *prevalent* delirium represent patients with ongoing delirium upon sampling during spinal anesthesia. Mann-Whitney U test, calculating the Gini coefficient based on the area under the curve ((2*Area under the curve)-1). The Gini coefficient is here comparable for all variables. The subsequent scale (-1 to 1) represents effect sizes that can be considered weak (≥ 0.1 to <0.3), moderate (≥ 0.3 to <0.4), and strong (≥ 0.4). *$P$-value < 0.05, **$P$-value < 0.001.

S. Fig 1

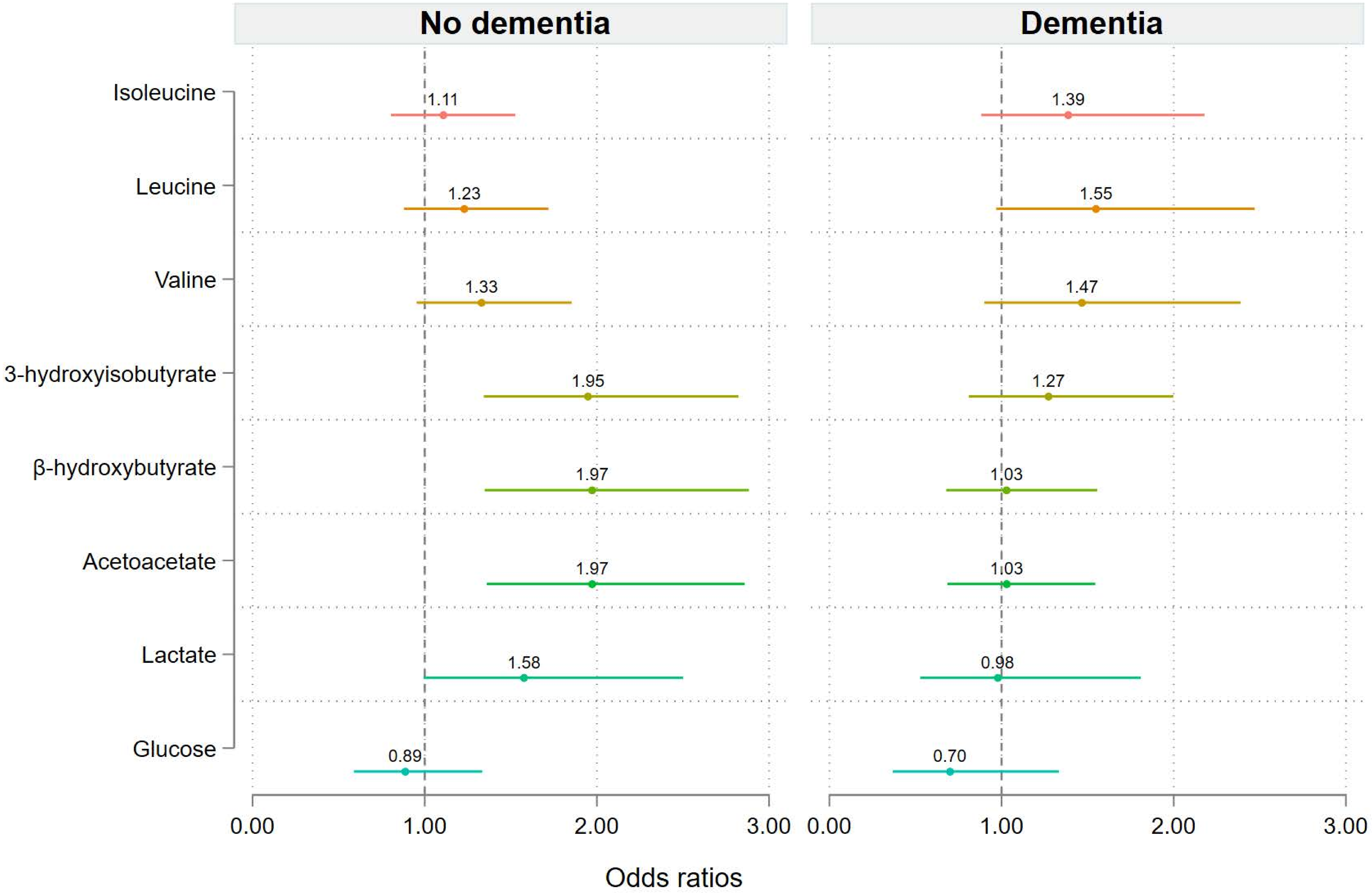

No dementia
Dementia
Isoleucine
Leucine
Valine
3-hydroxyisobutyrate
β-hydroxybutyrate
Acetoacetate
Lactate
Glucose
1.11
1.23
1.33
1.95
1.97
1.97
1.58
0.89
1.39
1.55
1.47
1.27
1.03
1.03
0.98
0.70
0.00
1.00
2.00
3.00
Odds ratios

S. Fig 2

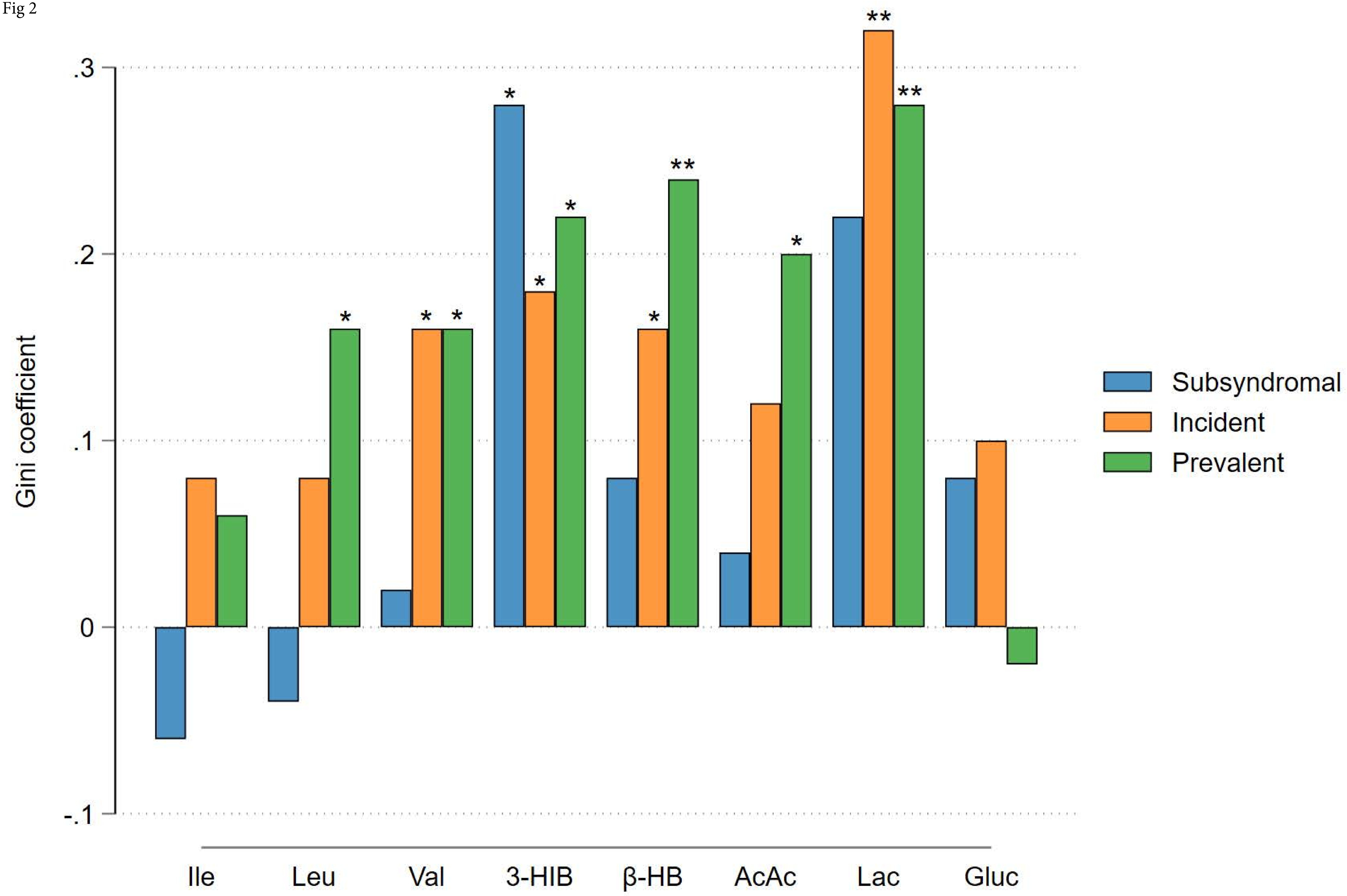

Gini coefficient
.3
.2
.1
0
-.1
*
*
*
*
*
*
*
*
**
*
**
**
Ile
Leu
Val
3-HIB
β-HB
AcAc
Lac
Gluc
Subsyndromal
Incident
Prevalent
