# Supplementary material for: Impaired glucose utilization in the brain of patients with delirium following hip fracture
Source: Brain. 2023 Sep 2;147(1):215–23. doi: 10.1093/brain/awad296 (PMC10766236; doi:10.1093/brain/awad296)
Supplement: awad296_Supplementary_Data [file awad296_supplementary_data.zip › brain-2022-02257-File008.pdf]

Author contributions

Irit Titlestad: Revision of manuscript and interpretation of the data. Data collection regarding Diabetes Mellitus diagnosis and plasma glucose. Preparation of manuscript.

Leiv Otto Watne: Initiation and design of the study. Data collection from all cohorts at all sites. Interpretation of the data and revision of manuscript. Preparation of manuscript.

Gideon A. Caplan: Initiation and design of the study. Interpretation of the data and revision of manuscript.

Adrian McCann: Initiation and design of the study. Biomarker analyses in serum and CSF at Bevital. Interpretation of the data and revision of manuscript.

Per Magne Ueland: Biomarker analyses in serum and CSF at Bevital. Interpretation of the data and revision of manuscript.

Bjørn Erik Neerland: Data collection, including delirium diagnostics, in hip fracture cohort 1 and 2. Interpretation of the data and revision of manuscript.

Marius Myrstad: Data collection at Bærum Hospital. Interpretation of the data and revision of manuscript.

Nathalie Bodd Halaas: Data collection in cohort of cognitively unimpaired adults. Interpretation of the data. Preparation of manuscript.

Christian Pollmann: Data collection at Akershus University Hospital. Interpretation of the data and revision of manuscript.

Kristi Henjum: Biomarker analyses (glucose and lactate). Interpretation of the data and revision of manuscript.

Anette Hylen Ranhoff: Data collection at Diakonhjemmet. Interpretation of the data and revision of manuscript.

Lene B. **Solberg:** Data collection at Oslo University Hospital. Interpretation of the data and revision of manuscript.

Wender Figved: Data collection at Bærum Hospital. Interpretation of the data and revision of manuscript.

Colm Cunningham: Initiation and design of the study. Interpretation of the data and revision of manuscript.

Lasse M. Giil: Planning of the study. Statistical analysis and interpretation of the data. Preparation of manuscript.
